# Magnetic Molecularly Imprinted Polymers for Selective Extraction of Aflatoxins from Feeds

**DOI:** 10.3390/toxins16030120

**Published:** 2024-02-29

**Authors:** María del Carmen Pérez-Álvarez, Natalia Arroyo-Manzanares, Natalia Campillo, Pilar Viñas

**Affiliations:** Department of Analytical Chemistry, Faculty of Chemistry, Regional Campus of International Excellence “Campus Mare Nostrum”, University of Murcia, 30100 Murcia, Spain; mariacarmen.pereza2@um.es (M.d.C.P.-Á.); ncampi@um.es (N.C.); pilarvi@um.es (P.V.)

**Keywords:** aflatoxins, feeds, magnetic molecularly imprinted polymers, magnetic separation technology, HPLC-FLD

## Abstract

Magnetic molecularly imprinted polymers (MMIPs) have fused molecular imprinting technology with magnetic separation technology, emerging as an innovative material capable of recognizing specific molecules and efficiently separating target substances. Their application to the extraction and purification of mycotoxins has great potential, due to the toxicity and economic impact of these contaminants. In this work, MMIP has been proposed as a sample treatment for the determination of main four aflatoxins (B_1_, B_2_, G_1_ and G_2_) in pig feed. The MMIP was formed through the integration of magnetic material (Fe_3_O_4_) with commercial molecularly imprinted polymers, avoiding the synthesis step and, therefore, simplifying the process. The analyses were carried out by high-performance liquid chromatography with fluorescence detection and the method was validated and limits of quantification (LOQs) between 0.09 and 0.47 ng/g were obtained, below the allowed or recommended levels by the European Union. Repeatability and intermediate precision showed relative standard deviations lower than 10% in all cases and trueness ranged from 92 to 111%. Finally, the proposed method was applied to 31 real pig feed samples, detecting aflatoxins with concentrations between 0.2 and 3.2 ng/g.

## 1. Introduction

Magnetic molecularly imprinted polymers (MMIPs) are formed through the integration of inorganic nanoparticles with molecularly imprinted polymers (MIPs). These MMIPs are developed in a core–shell configuration, where nanoparticles constitute the core, while selective polymeric layers serve as the shell enveloping the core surface. Essentially, MMIPs embody a synthesis of the optimal features from both inorganic nanoparticles and MIPs. The specificity of cavities imprinted on the MIP combines with the superparamagnetic attributes of nano-magnetite, which facilitates the extraction process of various analytes in complex matrices [1].

Recently, MMIPs have attracted wide attention since possess several promising characteristics in comparison with other media used for separation and adsorption [2]. These sorbents offer extensive surface areas, excellent biocompatibility, easy handling and straightforward customization. The inherent magnetic responsiveness of MMIPs establishes them as a potent and effective approach for streamlining the pre-concentration, separation and manipulation of analytes, particularly in large-scale operations. Because they can be effortlessly isolated from the sample matrix using an external magnetic field, the need for high-speed centrifugation and additional filtration is obviated when employing MMIPs as affinity adsorbents [3]. Some key applications include environmental contaminant extraction from soils and water, analysis of food contaminants, sample preparation in clinical diagnostics, water quality monitoring, detection of drugs in biological samples and forensic analysis of illicit substances. MMIPs enhance the efficiency and selectivity of analytical processes in various fields, showcasing their versatility in addressing complex sample matrices and the selective extraction of important contaminants such as aflatoxins (AFs).

AFs are toxic secondary metabolites produced by some species of *Aspergillus fungi*, especially *A. flavus* and *A. parasiticus*. They can be produced before, but especially after, harvest and can contaminate a variety of crops, including cereals, nuts, spices as well as feed [4]. Within AFs, about 30 compounds have been identified, the main ones being AFs B_1_, B_2_, G_1_ and G_2_. These mycotoxins are thermostable compounds that can persist during the milling, washing and processing of foodstuffs so that their content is not reduced in the final products.

AFs are recognized as genotoxic carcinogens by the Scientific Committee on Food [5]. Their descending order of toxicity is as follows: AFB_1_ > AFG_1_ > AFB_2_ > AFG_2_. Notably, AFB_1_ has been established as carcinogenic in all experimental animals and has held the classification as a human carcinogen by the World Health Organization (WHO) since 1988. As a consequence, the International Agency for Research on Cancer (IARC) has categorized AFB_1_ as a Group 1 substance, indicating substantial evidence of its carcinogenicity in humans [6].

Contamination by AFs can affect animal and human health and cause various adverse effects. In the case of animal feed, only AFB_1_ is regulated by European legislation, the maximum content fixed is 20 ng/g for pig, poultry and ruminant feed, 10 ng/g for calves and lambs and, for milk-producing animals and young animals, the limit is set at 5 ng/g [7]. In addition, codes of good hygienic practice are also established in the production and storage chain in order to achieve a reduction in the exposure of the population.

Compound feeds usually contain a variety of ingredients, such as grains, feed by-products, minerals and additives. The diversity of components can complicate the identification and quantification of AFs during the analysis, due to the high number of interferents implying a significant matrix effect. In addition, the composition of feed can vary considerably depending on the brand, batch and geographic region. This variability makes it difficult to establish standardized analysis methods and interpret the results. Therefore, very selective extraction methods are required where MMIPs could have great potential.

A recent review summarizes studies that applied advanced MMIPs for the analysis of mycotoxins in food [2]. This novel sample treatment has been applied for the extraction of AFs [8,9] and other mycotoxins, such as ochratoxins [10], sterigmatocystin [11], patulin [12,13] and zearalenone [14,15,16,17,18], mainly in cereals (wheat, corn, rice, millet and coix lacryma-jobi), but also in wine, soy sauce, vinegar or juice.

The most frequently used magnetic material is Fe_3_O_4_ [12,14,16,17,19,20], although applications have also been described magnetically modified hydroxyapatite (Fe_3_O_4_-HAP) [18] or the use of Fe_3_O_4_ as the magnetic core with the addition of chitosan (CS) and SiO_2_, to improve the biocompatibility, stability and dispersibility of the MIP adsorbent and graphene oxide (GO), since is an excellent supporting material for synthesizing MIP (Fe_3_O_4_@SiO_2_@CS-GO) [15]. Magnetic halloysite nanotubes [13], magnetic nanofibrous and porous carbon microsphere [10], or a magnetic core of Co_3_O_4_ with nanoporous carbon [11], have also been proposed. Regarding the template used for the generation of MIPs, 5,7-dimethoxycoumarin or ethyl 3-coumarincarboxylate have been used for the aflatoxin-specific MMIPs.

In this work, the potential of MMIPs for the extraction and preconcentration of AFs in feed samples continues to be explored. To the best of our knowledge, this is the first time that these sorbents have been applied for the determination of mycotoxins in these complex matrices.

## 2. Results and Discussion

### 2.1. Optimization of Sample Treatment

Although the use of MMIPs has multiple advantages over other sample treatment methods, the synthesis of MIPs can complicate the procedure, and sometimes the use of expensive templates is required to achieve effective recognition of the analyte molecule. Therefore, in this study, the use of commercial MIPs has been chosen, although it has the disadvantage that its composition and the template used for its preparation are not known. The MIPs utilized are commercially available as solid phase extraction (SPE) cartridges. The proposal involves magnetizing these MIPs and utilizing them in a procedure based on magnetic dispersive micro-solid phase extraction, which offers several practical advantages over traditional SPE, including faster extraction times, reduced sample volumes and improved efficiency in handling complex sample matrices. The preparation procedure for the MMIPs is described in Section 4.3.

**Figure 1:**
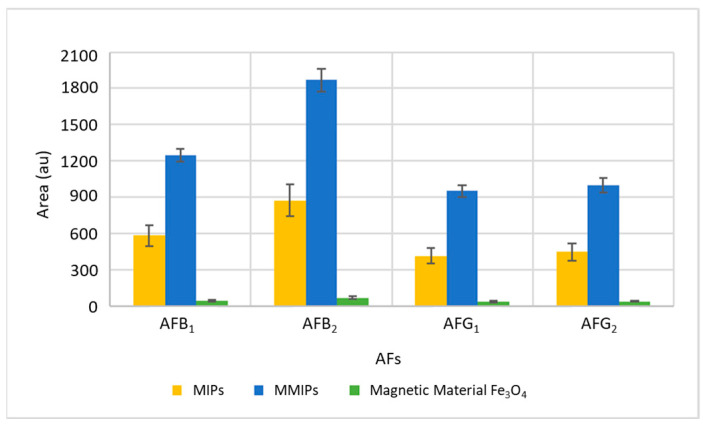
Comparison of the extraction efficiency of MMIPs, magnetic material without MIPs coating and MIPs. The bars show the area obtained for each AF in each experiment and error bars indicate the maximum and minimum of the replicas.

To verify the effectiveness of MMIPs to extract and preconcentrate AFs, they were applied and compared with the direct use of the magnetic material Fe_3_O_4_ without MIPs and the use of the commercial MIPs using the same procedure but, in this last case, introducing a centrifugation process for the isolation of the MIPs. For this purpose, a pig feed sample free of AFs was used, which was spiked at a concentration of 100 ng/g. The results are shown in Figure 1. As can be seen, in addition to their multiple advantages, MMIPs considerably improve the efficiency of extraction. MMIPs exhibit the selectivity of MIPs and as they are prepared by the polymerization of functional monomers in the presence of a molecular template and when the template is removed, MIPs have specific binding sites that confer molecular recognition to the polymer, having therefore a high affinity to aflatoxins than the magnetic material.

The binding of MIPs to magnetic particles facilitates the isolation and recovery of the analytes of interest with respect to the rest of the analytes present in the matrix using a magnetic field, which can be a useful tool for the analysis of complex samples. Magnetic property reduces extraction steps and therefore prevents sorbent losses improving recovery compared to using only MIPs.

In order to optimize the procedure based on magnetic dispersive micro-solid phase extraction, both absorption and desorption efficiency were evaluated, and the amount of mass of MMIPs, sample amount, the addition of salt to promote extraction, adsorption and desorption times and nature and volume of desorption solvent were investigated. The optimal values were selected based on the highest extraction recovery, i.e., the largest area obtained for the four AFs.

The optimization of the parameters associated with the adsorption (amount of MMIPs, adsorption time and salt addition) was carried out using a multivariate study because these parameters are closely related. A central composite design (2^3^ + star, face centered), with three spaced central points, involving 17 runs, was used as an approach to generate the response surface, using the area as an analytical response. The different factors were studied in the following ranges: amount of MMIPs (10–50 mg); adsorption time (1–20 min); and percentage of NaCl (0–10% *m*/*v*). The correlation coefficients R2 were in the range of 70.7 to 93.5%, which proves the suitability of the experiment. According to the Pareto diagrams, all variables were significant for all or some of the mycotoxins: the adsorption time significantly influenced the extraction of four AFs, while salt content and mass MMIPs were significant in the extraction of AFB_1_ and AFB_2_, and AFB_1_ and AFG_1_, respectively.

**Figure 2:**
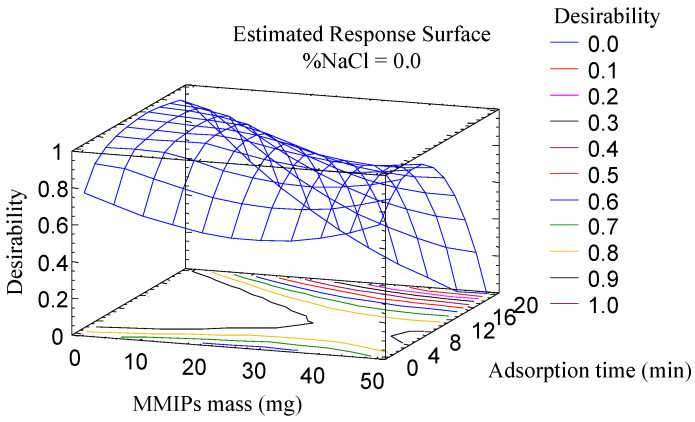
Estimated response surface for the study of significant variables using MMIPs by multiple response optimization. This optimization was based on a central composite design (2^3^ + star, face centered), with three spaced central points, involving 17 runs, which was used as an approach to generate the response surface, using the area as an analytical response.

The desirability function (Figure 2) was used for multiple response optimization resulting in the following optimum values: 10 mg of MMIPs, an adsorption time of 10 min and 0% of NaCl.

To optimize the desorption stage, the nature of the desorption solvent was initially investigated, and the following solvents were studied: acetonitrile (ACN), methanol (MeOH), ethyl acetate, ethanol (EtOH), acetone and methyl isobutyl ketone. All solvents gave rise to similar desorption efficiencies, and ethyl acetate was therefore selected as optimal since it allowed a subsequent rapid drying step, allowing reconstitution in a smaller volume, thereby improving the preconcentration factor of the sample treatment process.

**Figure 3:**
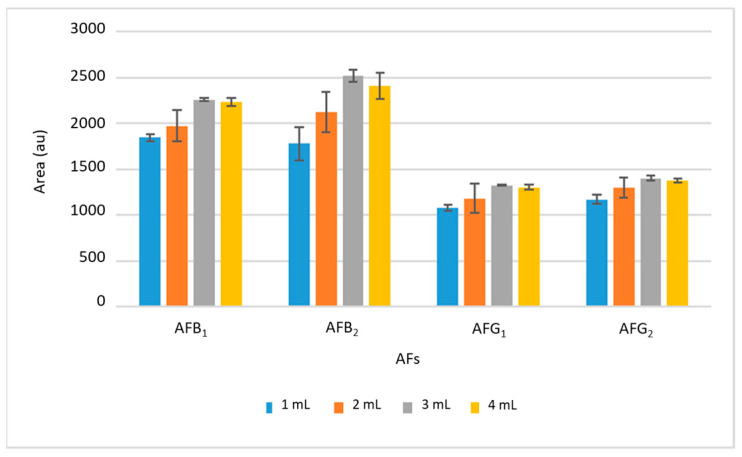
Optimization of desorption solvent volume. The bars show the area obtained for each AF in each experiment and error bars indicate the maximum and minimum of the replicas.

Subsequently, the volume of ethyl acetate was studied in the range between 1 and 4 mL. The results are shown in Figure 3, where it can be seen that the intensity of the signals increased with the desorption volume up to 3 mL; from this volume, the signal was practically maintained, so larger volumes will not improve the extraction efficiency and 3 mL was chosen as optimal. The desorption time was also studied between 2 and 15 min, selecting 10 min as optimal because no significant differences were found using longer times.

**Figure 4:**
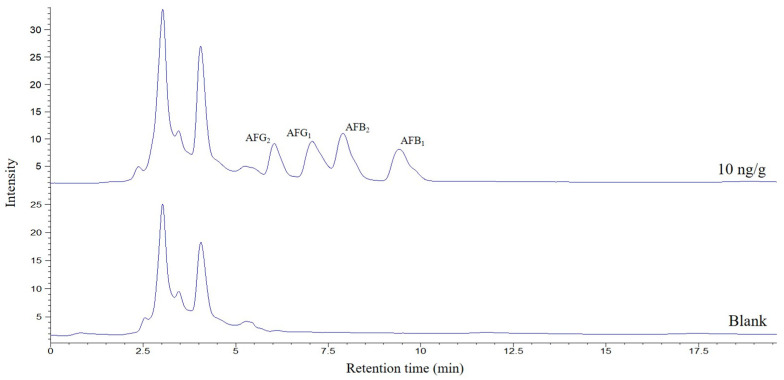
Chromatograms obtained from MMIPs isolation and HPLC-FLD analysis under the finally selected conditions of a pig feed sample spiked at 10 ng/g and a blank sample.

Drying the desorption solvent and reconstituting it in a smaller volume would improve the preconcentration factor, although it would also increase the matrix effect. To evaluate this process, reconstitution volumes between 250 and 500 μL using a mixture of MeOH/water (50:50, *v*/*v*) were studied. A volume of 250 μL was selected for further experiments, the minimum that allows good filtering and handling of the extract. Finally, and again looking for better preconcentration factors, the sample quantity was investigated between 0.5 and 2 g. It was found that increasing the mass, the areas of the AFs increased; however, above 1.5 g, interferences began to be observed in the chromatogram that worsened the selectivity of the method, so this amount was selected as optimum. A typical chromatogram corresponding to a spiked pig feed sample submitted to the proposed method is shown in Figure 4.

### 2.2. Validation of the Analytical Method

To validate the method, linear dynamic ranges, the limit of detection (LOD) and quantification (LOQ), precision and trueness were evaluated. For this, blank samples were used (previously analyzed to establish the absence of detectable AFs) that were spiked with the different analytes. Table 1 shows the results of this validation.

To assess the linearity of the method, blank samples were spiked with the different analytes. In total, six different concentration levels between 0.5 and 100 ng/g were included, and the analytical procedure was performed in duplicate at each concentration. Satisfactory determination coefficients (R^2^ > 0.99) were obtained, confirming that all analytical responses were linear over the studied ranges. The LODs and LOQs were determined as the minimum concentration of AFs in the spiked blank samples with a signal-to-noise ratio of 3 and 10, respectively. In this case, LOD varied between 0.03 and 0.14 ng/g, and the LOQ was between 0.09 and 0.47. The proposed methodology allows obtaining LOQs lower or similar to those described for the determination of AFs in feed using HPLC-FLD [19,20,21].

The precision of the method was studied in terms of repeatability on the same day and between days. For this purpose, spiked feed samples were analyzed at three different concentration levels (0.5, 10 and 50 ng/g). Relative standard deviations (RSD) were lower than 10%.

Finally, the trueness of the method was studied using recovery studies and the analysis of a reference material. For the recovery study, fortification of blank samples was carried out at three concentration levels (0.5, 10 and 50 ng/g). The observed signal was plotted against the actual concentration. The measured concentration was determined using the obtained calibration curves, and the recovery was calculated as 100 × measured concentration/actual (added) concentration. Recoveries were between 91.7 and 110.9% in all the cases, fulfilling the current legislation [22].

As previously indicated, the method was also validated using certified reference material for AFs in feed, which was cereal-based and supplied in a finely milled form. The proposed method was applied for the analysis of this reference material and the results are shown in Table 2. In addition, to ensure the suitability of the method, the certified material was also analyzed using the same sample treatment and ultra-high-performance liquid chromatography with high-resolution mass spectrometry (UHPLC-HRMS) analysis to ensure the identification of the compounds. A total of three experimental replicates were analyzed. The results were compared using the one-way repeated measures analysis of variance test. No significant differences were found with a *p*-value greater than 0.05 (*p* = 0.1978), which demonstrates the validity of the developed method.

### 2.3. Analysis of Feed Samples

The validated method was applied for the analysis of 31 real pig feed samples, which were analyzed in duplicate and the obtained mean value and standard deviation are shown in Table 3.

AFB_1_ and AFB_2_ were present in 96.8% of the samples and AFG_2_ in 80.6%. In contrast, none of the samples showed concentrations above the LOD for AFG_1_. AFB_1_ is the one with the highest concentrations ranging between 0.24 and 3.2 ng/g, followed by AFB_2_ ranging between 0.2 and 1.3 ng/g and, finally, AFG_2_ with values below 0.78 ng/g. Compared to the legislated levels, it is found that all samples comply with the requirements for maximum AFB_1_ content [8]. In addition, in all samples, more than one AF was detected. Specifically, 26% of the analyzed samples present cooccurrence of 2 AFs, and 74% of samples presented 3 of the studied AFs.

## 3. Conclusions

Magnetic separation technology based on the use of MMIPs has shown to have great potential to determine AFs in feed, a matrix that today represents a great challenge due to the variety of its ingredients and therefore its high matrix effect. The proposed MMIPs have allowed the extraction and purification of AFs in an excellent manner, allowing LOQs below the current limits established in the legislation. The union of the MIPs to the magnetic material provides a simplification of the analysis method, avoiding the use of SPE, reducing volumes of solvent and fulfilling the principles of Green Analytical Chemistry. In addition, the use of already commercial MIPs also helps to simplify sample treatment because the synthesis process is also avoided, which allows for reducing uncertainty and obtaining a very exact (truthful and precise) method. The application of the method for the analysis of 31 real pig feed samples has shown that in all samples more than one AF was detected, AFB_1_ was the one with the highest concentrations ranging between 0.24 and 3.2 ng/g, but in all cases, the limits established by legislation were not exceeded.

## 4. Materials and Methods

### 4.1. Chemicals and Reagents

MeOH, ACN, ethyl acetate, acetone, EtOH and methyl isobutyl ketone were obtained from Sigma Aldrich (St. Louis, MO, USA). Commercial Affinisep MIPs (selective SPE cartridges format 6 mL-200 mg for AFs, Ochratoxin A, HT-2, T-2, Fumonisins, Zearalenone and Deoxynivalenol), Mohr’s salt, iron trichloride hexahydrate and ammonium hydroxide were also supplied by Sigma-Aldrich and used to prepare the MMIPs. A certified reference material (ERM-BE376 compound feedingstuff) was obtained from Sigma-Aldrich and was used for method validation.

The water used for the analyses was purified using a Milli-Q system (Millipore, Bedford, MA, USA).

Individual standards of AFs (AFB_1_, AFB_2_, AFG_1_ and AFG_2_) at 1 mg (98% purity) were obtained from Sigma-Aldrich. Solutions of each AF at 1000 μg/mL in ACN were prepared and stored at −20 °C. Intermediate dilutions of the AFs mixture were prepared weekly at 10 μg/mL in ACN.

### 4.2. Instrumentation and Software

The instrument used was an Agilent 1100 HPLC system (Agilent, Waldbronn, Germany), equipped with a quaternary pump (G1311A) and coupled to two detectors, one diode array (G4212B) and one fluorescence detector (G1321A). The HPLC and detectors are connected to a UVE photochemical reactor (LCTech, Obertaufkirchen, Germany). The chromatographic column used was an ACE Excel 3 C18 (15 cm × 4.6 mm, 3 μm), made of high-purity silica by Advanced Chromatography Technologies (Aberdeen, Scotland).

For UHPLC-HRMS analysis, an Agilent 1290 Infinity II Series HPLC (Agilent Technologies, Santa Clara, CA, USA) with a high-speed binary pump coupled to an Agilent 6550 QTOF mass spectrometer and an Agilent dual jet stream electrospray ionization source (AJS-Dual ESI) was used. Agilent Technologies MassHunter software (Version B.07.00, Santa Clara, CA, USA) was used to obtain the data.

For sample treatment, an IKA-KS-130 Basic Orbital Shaker (Staufen, Germany) and a Horizon Technology Xcelvap air-drying system (Salem, MA, USA) were used. The permanent magnets used were blocks made of Nd-Fe-B with a strength of 33 kg, a weight of 86 g and dimensions of 50 × 15 × 15 mm obtained from Supermagnete (Gottmadingen, Germany). For sample filtration, 0.22 μm × 25 mm nylon syringe filters obtained from Agela Technologies (New York, NY, USA) were used.

The statistical tools used for data processing were Microsoft Office Excel version 16.82 (Microsoft, Redmond, WA, USA), SigmaPlot 13.1 (Systat, Software Inc., San Jose, CA, USA) and Statgraphics (Statistical Graphics, Rockville, MD, USA).

### 4.3. Preparation of MMIP

To obtain the MMIPs, 0.85 g of Mohr’s salt and 0.42 g of iron trichloride hexahydrate were weighed and dissolved in 250 mL of Milli-Q water. Then, 0.5 g of commercial MIPs were added and heated at 50 °C for 20 min in an ultrasonic bath. After this time, 25 mL of 8 M ammonium hydroxide was added dropwise, keeping the temperature at 50 °C. The mixture was kept in the bath without ultrasounds at 50 °C for another 30 min. Finally, the MMIPs were collected with the help of a magnet and washed twice with Milli-Q water and twice with EtOH. They were left to dry at 70 °C and, once dry, were stored in glass vials at room temperature until further use.

### 4.4. Samples

A total of 31 pig feed samples were analyzed, all from the region of Murcia (Spain). In addition, one pig feed sample free of AFs was used for the optimization and validation of the method. All samples were stored at room temperature until analysis.

### 4.5. Sample Treatment

The treatment consisted of weighing 1.5 g of feed sample into a 15 mL centrifuge tube, adding 5 mL of Milli-Q water and then shaking for 20 s. Then, 10 mg of the MMIPs were added. The mixture was orbitally shaken for 10 min, the MMIPs were removed with the help of a magnet and the supernatant was decanted. For desorption of the analytes, 3 mL of ethyl acetate was added and orbitally shaken again for 10 min. After this time, with the help of an external magnet, the MMIPs were also removed and the supernatant solution was collected, placed in a test tube and dried completely with the help of an air stream at 35 °C. After drying, the sample was reconstituted by adding 250 μL of a MeOH: water mixture (50:50, *v*/*v*). Finally, the extract was filtered through a 0.22 μm nylon filter into a 2 mL vial with a 250 μL insert and analyzed by HPLC-FLD.

### 4.6. HPLC-FLD Analysis

The separation was carried out on an ACE Excel 3 C18 column (15 cm × 4.6 mm, 3 μm). The mobile phase consisted of a mixture of Milli-Q water, MeOH and ACN in a 65:20:15 (*v*/*v*/*v*) ratio, respectively, working in isocratic mode. The flow rate of the mobile phase was set at 1 mL/min and the injection volume of 20 μL. In the fluorescence detection system, 360 nm was selected as the excitation wavelength and 440 nm as the emission wavelength.

### 4.7. UHPLC-HRMS Analysis

For validation, a UHPLC was used, using a column ZORBAX RRHD Eclipse Plus C18 column (10 cm × 2.1 mm, 1.8 μm particle size) and a 0.3 μm inline filter, both from Agilent Technologies. The mobile phase used was composed of solvent A (water/MeOH (95:5, *v*/*v*)) and solvent B (MeOH/water (95:5, *v*/*v*)). Both solvents contained 0.3% formic acid and 5 mM ammonium formate. The gradient profile was as follows: 0–0.5 min 40% B, 0.5–5.5 min 40–70% B, 5.5–6 min 70–40% B and 6–8 min 40% B at a flow rate of 0.4 mL/min. The sample volume injected was 20 μL. Positive mode operation was used in the mass spectrometer. The drying gas flow rate was 16 L/min at a temperature of 130 °C and a nebulizer gas pressure of 30 psi. The enveloping gas flow rate was 11 L/min and the temperature was 300 °C. For data acquisition, MS scans were configured in the range of 100–1000 *m*/*z*. Three collision energies (0, 10 and 40 V) were used in each cycle.

## Figures and Tables

**Table 1:** Method characterization data for AFs determination in feeds.

AFs	Equation (x, ng/g)		R^2^	LOD (ng/g)	LOQ (ng/g)	
AFB_1_	y = 19.28x − 0.2953		0.9999	0.0711	0.237	
AFB_2_	y = 23.45x + 2.291		0.9993	0.0594	0.198	
AFG_1_	y = 13.16x + 22.96		0.9949	0.0273	0.091	
AFG_2_	y = 12.34x − 1.317		0.9993	0.1425	0.475	
	**Intra-day precision, RSD (%) (n = 9)**			**Inter-day precision, RSD (%)** **(n = 9)**		
	0.5 ng/g	10 ng/g	50 ng/g	0.5 ng/g	10 ng/g	50 ng/g
AFB_1_	3.5	4.1	3.1	9.6	4.4	4.5
AFB_2_	2.4	4.4	5.2	8.3	4.9	5.4
AFG_1_	7.2	9.3	3.3	8.7	9.7	4.6
AFG_2_	6.9	3.7	3.8	7.3	9.0	4.6
	**Trueness (RSD, %)**					
	0.5 ng/g		10 ng/g		50 ng/g	
AFB_1_	102.7 (4.0)		100.1 (1.3)		110.1 (2.4)	
AFB_2_	110.9 (4.7)		105.5 (1.7)		103.8 (3.2)	
AFG_1_	107.0 (3.9)		107.0 (5.7)		110.4 (2.8)	
AFG_2_	91.7 (5.0)		96.7 (4.8)		99.8 (2.7)	

**Table 2:** Analysis of the reference material by UHPLC-HRMS and HPLC-FLD (concentration expressed in ng/g).

AFs	Certificated Concentration *	Concentration Obtained by HPLC-FLD	Concentration Obtained by UHPLC-HRMS
AFB_1_	12.9 ± 1.8	13.8 ± 0.6	14.6 ± 0.7
AFB_2_	0.68 ± 0.1	0.58 ± 0.08	0.73 ± 0.09
AFG_1_	5.2 ± 0.8	5.6 ± 0.3	5.8 ± 0.3
AFG_2_	----	ND	ND

* The certified reference material included only aflatoxins B1, B2, G1 and G2. ND: not detected.

**Table 3:** Analysis of real samples with the proposed methodology (concentration expressed in ng/g).

Sample	AFB_1_	AFB_2_	AFG_1_	AFG_2_
M1	0.43 ± 0.05	<LOQ	ND	<LOQ
M2	0.24 ± 0.08	0.20 ± 0.04	ND	<LOQ
M3	<LOQ	ND	ND	<LOQ
M4	0.53 ± 0.04	<LOQ	ND	ND
M5	0.278 ± 0.001	<LOQ	ND	<LOQ
M6	0.25 ± 0.01	<LOQ	ND	ND
M7	0.75 ± 0.02	0.358 ± 0.005	ND	<LOQ
M8	3.2 ± 0.1	0.85 ± 0.06	ND	<LOQ
M9	0.58 ± 0.08	0.3 ± 0.1	ND	ND
M10	<LOQ	1.14 ± 0.08	ND	ND
M11	ND	0.69 ± 0.02	ND	<LOQ
M12	0.58 ± 0.02	0.69 ± 0.06	ND	<LOQ
M13	0.727 ± 0.009	0.9 ± 0.1	ND	ND
M14	0.68 ± 0.03	0.221 ± 0.006	ND	<LOQ
M15	0.62 ± 0.09	<LOQ	ND	<LOQ
M16	0.59 ± 0.09	<LOQ	ND	<LOQ
M17	0.46 ± 0.01	0.301 ± 0.002	ND	<LOQ
M18	<LOQ	1.2 ± 0.1	ND	0.52 ± 0.03
M19	0.313 ± 0.002	<LOQ	ND	ND
M20	<LOQ	0.82 ± 0.07	ND	<LOQ
M21	0.28 ± 0.05	<LOQ	ND	<LOQ
M22	1.8 ± 0.1	1.30 ± 0.02	ND	<LOQ
M23	0.7 ± 0.1	0.74 ± 0.04	ND	<LOQ
M24	<LOQ	0.63 ± 0.01	ND	0.78 ± 0.02
M25	<LOQ	0.86 ± 0.09	ND	<LOQ
M26	<LOQ	0.270 ± 0.003	ND	<LOQ
M27	<LOQ	0.6 ± 0.1	ND	<LOQ
M28	<LOQ	<LOQ	ND	<LOQ
M29	0.24 ± 0.04	<LOQ	ND	<LOQ
M30	0.29 ± 0.02	<LOQ	ND	<LOQ
M31	0.41 ± 0.03	<LOQ	ND	<LOQ

ND: not detected; <LOQ: value between the LOD and the LOQ.

## Data Availability

Data are contained within the article.
